# Ethanolic Fruit Extract of *Emblica officinalis* Suppresses Neuroinflammation in Microglia and Promotes Neurite Outgrowth in Neuro2a Cells

**DOI:** 10.1155/2021/6405987

**Published:** 2021-09-07

**Authors:** Sataporn Phochantachinda, Duangthip Chatchaisak, Piya Temviriyanukul, Anchana Chansawang, Pornsiri Pitchakarn, Boonrat Chantong

**Affiliations:** ^1^Prasu-Arthorn Animal Hospital, Faculty of Veterinary Science, Mahidol University, Salaya, Phutthamonthon, Nakhon Pathom 73170, Thailand; ^2^Department of Clinical Sciences and Public Health, Faculty of Veterinary Science, Mahidol University, Salaya, Phutthamonthon, Nakhon Pathom 73170, Thailand; ^3^Institute of Nutrition, Mahidol University, Salaya, Phutthamonthon, Nakhon Pathom 73170, Thailand; ^4^The Center for Veterinary Diagnosis, Faculty of Veterinary Science, Mahidol University, Salaya, Phutthamonthon, Nakhon Pathom 73170, Thailand; ^5^Department of Biochemistry, Faculty of Medicine, Chiang Mai University, Chiang Mai 50200, Thailand; ^6^Department of Pre-Clinical and Applied Animal Science, Faculty of Veterinary Science, Mahidol University, Salaya, Phutthamonthon, Nakhon Pathom 73170, Thailand

## Abstract

Inhibiting neuroinflammation and modulating neurite outgrowth could be a promising strategy to prevent neurological disorders. *Emblica officinalis* (EO) may be a potent agent against them. Although EO extract reportedly has anti-inflammatory properties in macrophages, there is limited knowledge about its neuroprotective activity by suppressing microglia-mediated proinflammatory cytokine production and inducing neurite outgrowth. The present study aimed to elucidate the effect of EO fruit extract on the lipopolysaccharide- (LPS-) induced neuroinflammation using microglial (BV2) and neuroblastoma (Neuro2a) cells. The results demonstrated that, in LPS-treated BV2 cells, EO fruit extract reduced nitric oxide, interleukin-6, and tumor necrotic factor-*α* production. It also enhanced the neurite length of Neuro2a cells, which was linked to the upregulation of TuJ1 and MAP2 expressions. In conclusion, these findings indicate that the ethanolic extract of EO fruits has promising neuroprotective potential to exhibit antineuroinflammation activity and accelerative effect on neurite outgrowth *in vitro*. Therefore, EO fruit extract can be considered a novel herbal medicine candidate for managing neuroinflammatory diseases.

## 1. Introduction

Neuroinflammation is the primary cause of most neurological diseases [1]. It is involved in major neurodegenerative disorders such as Alzheimer's disease (AD) and Parkinson's disease (PD) [2, 3]. Microglia are represented by resident immune cells in the defense system against inflammation in the brain [4]. Activated microglial and neuritic abnormalities constitute a significant AD and PD pathology [5, 6]. Neuroinflammation activates microglial cells to release proinflammatory cytokines and toxic factors, such as superoxide radicals, nitric oxide (NO), interleukin-6 (IL-6), and tumor necrotic factor-*α* (TNF*-α*), that damage neurons [7, 8].

In this study, lipopolysaccharide (LPS) application, as a potent stimulus of the microglial innate immune response [5], induced neuroinflammation associated with neurodegeneration, especially in the AD model [9, 10]. LPS can activate microglia to increase the production of inflammatory cytokines through both toll-like receptor 4 (TLR4) and mitogen-activated protein kinase (MAPK) pathways [11] that exacerbate the pathology of neurodegenerative processes [12]. The MAPK pathway is employed in cellular processes such as proliferation, differentiation, apoptosis, cell survival, cell motility, metabolism, stress response, and inflammation [13]. Activated microglia release proinflammatory cytokines (NO, IL-6, and TNF*-α*), inducing cognitive impairment and neuroinflammation [4, 5, 14, 15].

A previous study reported that interleukin-6 (IL-6) decreased the differentiation of neural stem/progenitor cells into neurons, promoted gliogenesis, oligodendrogliogenesis, and astrogliogenesis, and reduced neurogenesis via the MAPK/CREB pathway [16]. Moreover, inflammation-mediated neurodegeneration involves inducible nitric oxide synthase (iNOS) overexpression, causing toxicity to neurons by overproducing NO in activated microglia [17]. The MAPK pathway played a role in regulating several signaling molecules, including NO, IL-6, and TNF-*α* [18, 19]. Pieces of evidence indicate that suppressing the MAPK pathway can reduce neuroinflammation or neurodegenerative disorders [20–22]. Therefore, this study aimed to investigate neuroinflammation inhibition via the MAPK pathway suppression that would reduce inflammatory-related molecule production.

Differentiation of neuronal cells is an important key factor for the prevention of neurodegenerative diseases. Numerous specific neuronal markers are expressed during neuronal differentiation. Beta-III tubulin (TuJ1) is a marker for early differentiated neurons related to the cellular movement during mitosis [23]. Microtubule-associated protein-2 (MAP2) is a marker for mature neurons [24], which binds and stabilizes microtubules by altering their disassembly duration [25]. Many studies use TuJ1 and MAP2 as neuronal markers to investigate protein or mRNA expression [25, 26]. Both TuJ1 and MAP2 reportedly modulate the growth and stabilization of microtubules in neurites of Neuro2a cells [27]. Enhancing neurite outgrowth represented by their increased length and branching is essential for treating neurodegenerative diseases [28, 29]. Inducing neuronal differentiation was intended to be investigated in this study by measuring TuJ1 and MAP2 expression.

Using phytochemicals as nutraceuticals has expanded substantially. Natural products, used as nutraceuticals to increase pharmacological response and minimize some adverse effects, are extensively considered. Many traditional Ayurvedic medicinal plants such as *Bacopa monnieri* and *Mucuna pruriens* have been studied for either neuroprotective or anti-inflammatory properties in neuronal cells [30, 31]. The present study was focused on antineuroinflammation and neuroprotective effects of *Emblica officinalis* (EO) fruit ethanol extract. This species is found in many regions of Southeast Asia [32] and is widely accepted in Thailand as a traditional medicinal herb [33, 34]. EO crude extract contains various bioactive compounds, such as gallic acid (GA), ellagic acid (EA), ascorbic acid, mucic acid, tannin, quercetin, and rutin [35–37], which exerted protective activities, including anti-inflammatory, antitumor, antioxidation, and immunomodulatory effects, to prevent central nervous system diseases [34, 38, 39]. Previous studies suggested that both GA and EA might cross the blood-brain barrier. The present study, therefore, was concentrated on these two compounds. GA administration reportedly prevented oxidative damage and neuroinflammation during brain injury induced by LPS and ketamine administration in rats [40–42]. GA also protected memory and reduced long-term potentiation impairment caused by traumatic brain injury by reducing both brain lipid peroxidation and cerebral proinflammatory cytokines [43]. Furthermore, EA is known to attenuate anxiety, depression, locomotion behavior, brain edema, and inflammation in rats with cerebral ischemia/reperfusion by decreasing brain tissue inflammation [44]. EA showed a protective effect against brain injury induced by LPS and carbon tetrachloride in rats by modulating the Nrf-2, NF-*κ*B, and apoptotic pathways [45, 46]. Although EO's anti-inflammatory properties were previously investigated, the molecular basis behind these anti-inflammatory effects in the neuronal system has not yet been scrutinized. To the best of our knowledge, no studies have ascertained the effect of EO crude fruit extract on neuroinflammation using microglia and neurite outgrowth with neuroblastoma cells as model systems. Therefore, we intended to examine the role of EO fruit extract in regulating neuroinflammation and its neuroprotective effects in a cellular system made of microglia and neurons.

## 2. Materials and Methods

### 2.1. Reagents

A 2× qPCRBIO SyGreen 1-Step Lo-ROX kit was obtained from PCR Biosystems (Wayne, Pennsylvania, USA). We purchased the Bradford solution, blotting-grade blocker (nonfat dry milk), and polyvinylidene fluoride (PVDF) membrane from Bio-Rad (Benicia, CA, USA). All-*trans* retinoic acid, EA, GA, and LPS (from *Escherichia coli* O111 : B4) were acquired from Sigma-Aldrich (St. Louis, MO, USA). Antibodies specific to *β*-actin (cat no. 4970S), TuJ1 (cat no. 5568S), ERK1/2, (cat no.4695S), pERK1/2 (cat no. 4376S), JNK (cat no. 9252S), or pJNK (cat no. 4671S) came from Cell Signaling Technology (Danvers, MA, USA). We obtained cytosine arabinoside hydrochloride, the IL-6 quantitative sandwich enzyme-linked immunosorbent assay (ELISA) kit, radioimmunoprecipitation assay (RIPA) buffer, and XTT solution salt from Abcam (Cambridge, MA, USA). The TNF-*α* sandwich ELISA kit was purchased from BioLegend (San Diego, CA, USA). Ethanol and methanol were purchased from Merck (Darmstadt, Germany). Fetal bovine serum (FBS), fungizone, minimal essential medium (MEM), penicillin, Roswell Park Memorial Institute (RPMI) medium 1640, and streptomycin were acquired from GIBCO (Waltham, MD, USA). The Griess reagent and tetramethylbenzidine (TMB)-stabilized substrate for horseradish peroxidase were purchased from Promega (Madison, WI, USA). Tri-RNA Reagent was purchased from Favorgen (Kaohsiung, Taiwan).

### 2.2. EO Crude Fruit Extract Preparation

We purchased *Emblica officinalis* Gaertn. (EO) fruits from an orchard in Nakhon Pathom Province. The fruits were washed with tap water and rinsed with distilled water to remove potential contaminants. Then, clean fruits were chopped and dried in an oven at 60°C. We ground dried fruits into smaller parts for extraction. Next, we macerated EO fruit powder in 70% ethanol (at 1 : 10 w/v) at room temperature for six days (the powder was filtered and macerated in fresh 70% ethanol every two days). Ethanol was subsequently removed using a rotary evaporator (Heidolph, Wood Dale, IL, USA) at 40°C. We obtained EO crude fruit extract using a dry freezing machine (Modulyo, Waltham, MA, USA). Finally, we prepared stock solutions by dissolving the extract in dimethyl sulfoxide and stored at −80°C until further use.

### 2.3. Quantification of Compounds Present in EO Crude Fruit Extract by High-Performance Liquid Chromatography (HPLC)

We prepared EO crude fruit extract at the concentration of 1 mg/mL. GA and EA, used as standard compounds, were prepared at five concentrations (25, 50, 100, 200, and 400 *μ*g/mL). We filtered each solution by syringe through a nylon filter with a pore size of 0.45 *μ*m and subsequently subjected to HPLC analysis (Shimadzu High-Performance LC 10 AP; Shimadzu Scientific, MA, USA). Samples were eluted through an ACE Generix 5 *μ*m C18 column (4.6 mm × 250 mm) (Advanced Chromatography Technologies, Scotland, UK) using mobile phase: 0.1% acetic acid (A) and methanol (B) with a flow rate of 1.5 mL/min. The gradient program was as follows—0–15 min: 5% B; 15–40 min: 80% B; 40–42 min: 5% B; 42–50 min: 5% B. The injection volume of each sample was 10 *μ*L. A photodiode array detector detected eluted compounds, and the UV detection wavelength was set to 278 nm.

### 2.4. Cell Culture System

#### 2.4.1. BV2 Cell Culture

BV2 cells, a mouse microglial cell line, were purchased from Interlab Cell Line Collection (ATL033001). The cells were cultured at 37°C in RPMI medium 1640, containing 10% (v/v) heat-inactivated FBS, 100 IU/mL penicillin, 100 *µ*g/mL streptomycin, 0.25 mg/mL sodium bicarbonate, and 2.5 *µ*g/mL fungizone, in a humidified incubator containing 5% CO_2_. BV2 cells were seeded at a density of 1.5 × 10^3^ cells/cm^2^ in 75 cm^2^ flasks in RPMI medium with 10% FBS and then passed at approximately 80% confluence.

#### 2.4.2. Neuro2a Cell Culture and Differentiation

Neuro2a cells, a mouse neuroblastoma cell line, were purchased from ATCC (CCL-131TM). They were cultured at 37°C in MEM, supplemented with 10% FBS, 100 IU/mL penicillin, 100 *µ*g/mL streptomycin, 0.25 mg/mL sodium bicarbonate, and 2.5 *µ*g/mL fungizone, in a humidified incubator containing 5% CO_2_. Neuro2a cells were seeded at a density of 1.5 × 10^3^ cells/cm^2^ in a 75 cm^2^ flask in MEM medium with 10% FBS and then passed at approximately 80% confluence. After that, Neuro2a cells had to differentiate to express characteristics of neuron cells by retinoic acid before conducting further tests [47–49]. It was accomplished by seeding cells in DMEM with 2% FBS and changing medium every two days until six days passed. From day 0 to day 2, we used DMEM with 2% FBS and all-trans retinoic acid (10 *µ*M). After that, we used DMEM supplemented with 2% FBS, all-trans retinoic acid (10 *µ*M), and cytosine arabinoside hydrochloride (0.5 *µ*M/mL) from day 2 to day 4. After four days, the medium was replaced by DMEM containing 2% FBS and retinoic acid (10 *µ*M) until the end of the 6^th^ day.

### 2.5. Investigation of Toxicity of EO Fruit Extract and LPS to BV2 and Neuro2a Cells by XTT Reduction Assay

Neuro2a cells or BV2 cells were seeded in 96-well culture plates at a density of 1 × 10^4^ cells/well and incubated for 24 h in a CO_2_ incubator. The medium was subsequently replaced either by EO crude fruit extract resuspended in a medium (0.125–1 *µ*g/mL) or by LPS (1 *µ*g/mL) and further incubated for 24 h. After that, we removed the medium and added XTT solution to the cells. We performed further incubation at 37°C for 2 h. Finally, the absorbance was read at 450 nm using a microplate reader (BioTek, Winooski, VT, USA). We used untreated cells as a negative control. Data were expressed as a percentage compared to control.

### 2.6. Investigation of the Anti-Inflammatory Effect of EO Crude Fruit Extract on LPS-Induced Inflammation of BV2 and Neuro2a Cells

#### 2.6.1. Cell Treatment

We seeded either Neuro2a or BV2 cells in 6-well culture plates and allowed to adhere to the plate overnight in a CO_2_ incubator. We replaced the medium with EO (1 *µ*g/mL) or GA (31 ng/mL) or EA (15 ng/mL) in the presence of LPS (1 *µ*g/mL) and incubated for 24 h. GA and EA were used as positive controls.

#### 2.6.2. IL-6 and TNF-*α* Measurement by ELISA

After the treatment, the culture medium was collected and centrifuged at 2000× *g* for 10 min at 4°C. The resulting supernatant was subjected to ELISA. A quantitative sandwich ELISA kits was used to measure IL-6 and TNF-*α* in samples. The measurements were performed according to the manufacturer's recommendations. Concentrations of IL-6 and TNF-*α* were calculated from standards, ranging from 15 to 500 pg/mL.

#### 2.6.3. Nitric Oxide (NO) Measurement Using Griess Reagent

After the treatment, culture media of either Neuro2a or BV2 cells were collected and centrifuged at 2000× *g* for 10 min at 4°C. Nitrite concentration was assessed in the resulting supernatant using the Griess reagent, according to the manufacturer's recommendations. Briefly, 50 *µ*L of each culture medium was mixed with the same volume of the reagent. The nitrite level was determined using a microplate reader at the wavelength of 550 nm. Nitrite concentrations were calculated in comparison to a standard curve of sodium nitrite.

#### 2.6.4. Neurite Measurement and Morphology Determination

We treated Neuro2a cells with various GA or EA extract concentrations for 24 h. We observed the cells and photographed them under an inverted microscope (ECLIPSE Ts2; Nikon, NY, USA) with phase-contrast objectives. The length of neurites was measured using MicroCapture software v. 6.9.10. We conducted each experiment in triplicate.

For morphology imaging, we performed cell treatments in an imaging dish (TomoDish; Tomocube Inc., Daejeon, South Korea). Then, we directly observed the cells using an HT instrument (HT-2; Tomocube Inc., Daejeon, South Korea).

#### 2.6.5. Western Blot Analysis

After 24 h of treatment, we collected either Neuro2a or BV2 cells with a RIPA buffer. We measured protein concentration by Bradford assay. Each protein sample (10 *µ*g) was separated using 10% sodium dodecyl sulfate-polyacrylamide (SDS) gel, transferred to a PVDF membrane, and blocked with 5% (w/v) nonfat milk in Tris-buffered saline with 0.1% Tween 20 (TBST) at room temperature for an hour. The membrane was incubated overnight at 4°C with primary rabbit monoclonal antibodies against *β*-actin, TuJ1, ERK1/2, pERK1/2, JNK, and pJNK at the dilution of 1 : 1000. The membrane was further incubated at room temperature with secondary antibodies at 1 : 1000 dilution. The TMB substrate solution detected bands, and we analyzed their density by ImageJ software.

#### 2.6.6. RNA Extraction and Analysis by Reverse Transcription-Quantitative Polymerase Chain Reaction (RT-qPCR)

After the treatment, either Neuro2a or BV2 cells were collected and dissolved in Tri-RNA Reagent. Total RNA was extracted according to the manufacturer's instructions. The RT-qPCR reaction mixture contained 5 *μ*L of 2× qPCRBIO SyGreen 1-Step Lo-ROX, 0.5 *μ*L of 20× RTase GO, 0.4 *μ*L of each primer (10 *μ*M), 100 ng of RNA template, and 6.3 *μ*L of RNase-free water. PCRs were conducted in qTOWER3 Real-Time PCR (qPCR) Systems (Analytik Jena, Langewiesen, Germany). The cycle conditions included a reverse transcription step at 45°C for 10 min, initial polymerase activation step at 95°C for 2 min, and denaturation step of 40 cycles at 95°C for 5 s followed by a step of 60°C for 30 s. *GAPDH* was used as an internal control for normalization.  Table 1 presents the primer sequences.

### 2.7. Statistical Analysis

We performed statistical analysis using descriptive statistical procedures by GraphPad Prism v. 5 software. Values are presented as means ± SD from at least three independent experiments. Statistical analysis was performed using one-way ANOVA followed by Tukey's test. A probability value of 0.05 or less was considered to be statistically significant.

## 3. Results

### 3.1. GA and EA Contents of EO Crude Fruit Extract

**Figure 1:**
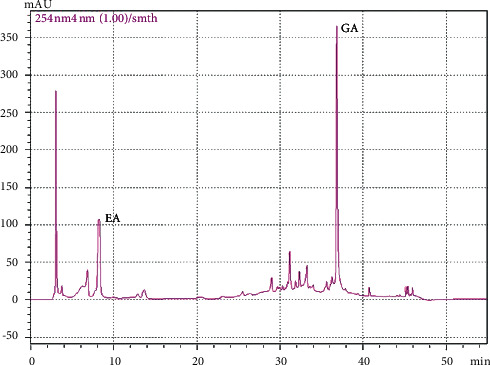
HPLC chromatogram of EO crude fruit extract. EA = ellagic acid, GA = gallic acid.

We obtained GA and EA contents from HPLC chromatograms. The maximum absorbance wavelength and retention time for GA and EA were 254 nm at 37 min and 278 nm at 8 min, respectively (Figure 1). EO crude fruit extract contained 31.15 mg/g dry weight GA and 14.99 mg/g dry weight EA.

### 3.2. Cytotoxicity of EO Crude Fruit Extract and LPS to BV2 and Neuro2a Cells

**Figure 2:**
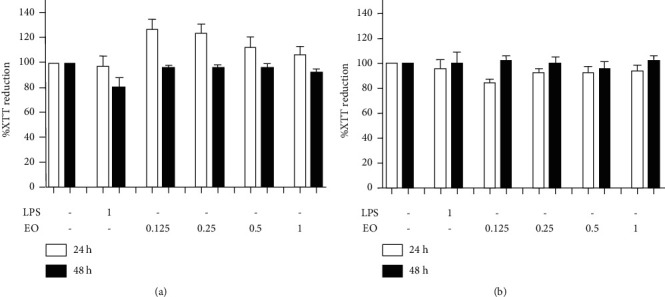
Cell viability after exposure to different EO crude fruit extract concentrations or LPS, determined by the XTT reduction assay. We treated the cells for 24 h or 48 h with varying concentrates of EO crude fruit extract (0.125, 0.25, 0.5, or 1 *μ*g/mL) or LPS (1 *μ*g/mL) before detecting cellular viability. The results are presented as mean ± SD. (a) BV2 cells and (b) differentiated Neuro2a cells.

We used murine BV2 cells and neuroblastoma Neuro2a cells as representative for neurons and microglia cells. BV2 and differentiated Neuro2a cells were exposed to EO crude fruit extract at the concentrations of 0.125, 0.25, 0.5, and 1 *µ*g/mL or LPS at 1 *µ*g/mL. After exposure, we determined cytotoxicity by the XTT reduction assay. None of the EO treatments showed decreased cellular viability after 48 h of exposure (Figure 2). However, 1 *µ*g/mL LPS treatment for 48 h was slightly toxic to BV2 cells as the %XTT reduction was lower than 80% (79.19%) (Figure 2(a)). Therefore, a suitable exposure time and a subtoxic concentration of LPS for the further experiments were estimated to be 24 h and 1 *µ*g/mL, respectively.

### 3.3. Effect of EO Crude Fruit Extract on LPS-Induced IL-6 and TNF-*α* Production in BV2 and Differentiated Neuro2a Cells

**Figure 3:**
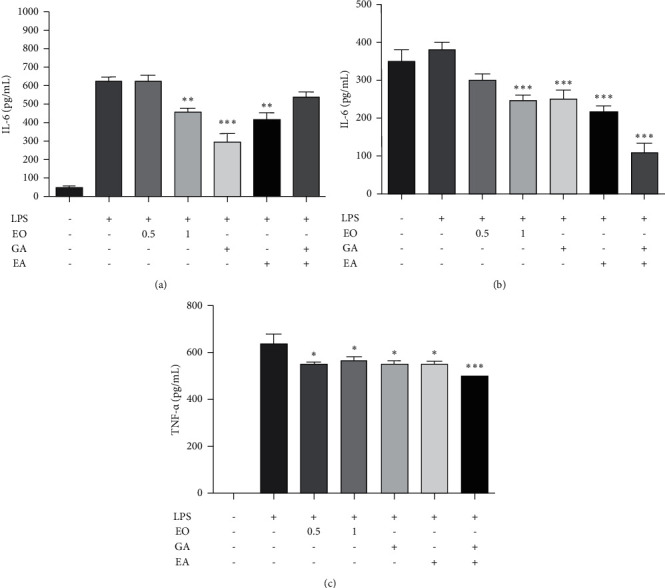
Effect of EO crude fruit extract on the proinflammatory mediator IL-6 and TNF-*α* in BV2 and differentiated Neuro2a cells. Both BV2 and differentiated Neuro2a cells were treated with LPS (1 *μ*g/mL) or EO crude fruit extract (0.5 and 1 *μ*g/mL). After 24 h, the supernatant was collected, and ELISA assessed the IL-6 and TNF-*α* levels. (a) IL-6 level in BV2 cells; (b) IL-6 level in differentiated Neuro2a cells; and (c) TNF-*α* level in BV2 cells ^*∗*^*p* < 0.05. (ANOVA; significant at ^*∗∗*^*p* < 0.01 and ^*∗∗∗*^*p* < 0.001, compared with the LPS-treated group.)

We used ELISA to evaluate the medium in which the LPS-induced BV2 and Neuro2a cells were grown and inflammatory cytokines' levels to examine the effects of EO crude fruit extract on LPS-stimulated IL-6 and TNF-*α* production.  Figure 3(a) shows that BV2 cells treated with 1 *µ*g/mL LPS showed significantly increased IL-6 production compared with those in the control group. A co-treatment with EO crude fruit extract at the concentration of 1 *µ*g/mL for 24 h significantly decreased the IL-6 production in a dose-dependent manner (*p* < 0.01). Moreover, differentiated Neuro2a cells could secrete IL-6 without LPS-induced inflammation. This result implicates that LPS could not induce IL-6 production in differentiated Neuro2a cells.  Figure 3(b) shows that EO crude fruit extract at 1 *µ*g/mL significantly decreased the baseline of IL-6 production in differentiated Neuro2a cells (*p* < 0.01). Consistently, BV2 cells treated with 1 *µ*g/mL LPS showed significantly increased TNF-*α* production compared with those in the control group. EO crude fruit extract at the concentrations of 0.5 and 1 *µ*g/mL significantly decreased production in the LPS-activated BV2 cells (*p* < 0.01) (Figure 3(c)). Furthermore, equivalent concentrations of GA and EA strongly reduced IL-6 and TNF-*α* production in BV2 and reduced the IL-6 level in differentiated Neuro2a cells. However, LPS was unable to induce TNF-*α* release in differentiated Neuro2a cells.

### 3.4. Effect of EO Crude Fruit Extract on LPS-Induced NO Production and iNOS mRNA Expression in BV2 and Differentiated Neuro2a Cells

**Figure 4:**
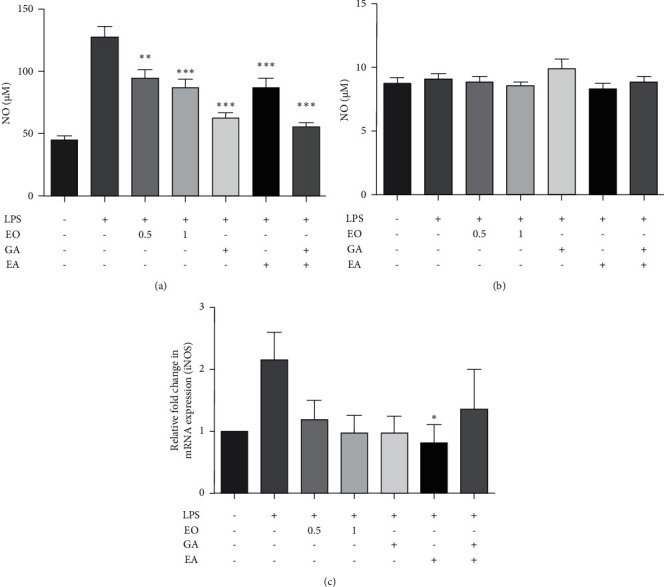
Effect of EO crude fruit extract on NO production and iNOS mRNA expression in BV2 and differentiated Neuro2a cells. Both BV2 and differentiated Neuro2a cells were treated with LPS (1 *μ*g/mL) or EO crude fruit extract (0.5 or 1 *μ*g/mL). After 24 h, the supernatant was collected, and NO levels were assessed using the Griess assay. Cells were collected to evaluate the iNOS mRNA expression levels. (a) BV2 cells, (b) differentiated Neuro2a cells, and (c) iNOS mRNA expression in BV2 cells were tested by RT-qPCR ^*∗*^*p* < 0.05 (ANOVA; significant at ^*∗∗*^*p* < 0.01 and ^*∗∗∗*^*p* < 0.001, compared with the LPS-treated group.)

To investigate the anti-inflammatory effect of EO crude fruit extract, we used LPS to stimulate NO production in BV2 cells and then measured its content using the Griess assay. The results in Figure 4(a) and 4(b) shows that EO crude fruit extract at the concentrations of 0.5 and 1 *µ*g/mL significantly decreased NO production in the LPS-activated BV2 cells in a dose-dependent manner (*p* < 0.01), but not in differentiated Neuro2a cells. In addition, the application of GA and EA at the concentration found in EO crude fruit extract (1 *µ*g/mL) successfully alleviated NO production in BV2 cells. Furthermore, we examined the effect of EO crude fruit extract at the concentrations of 0.5 or 1 *µ*g/mL on iNOS mRNA expression in BV2 cells. EO crude fruit extract tended to decrease the iNOS mRNA expression level by 44.67% and 54.61%, compared with the LPS-treated group. Nevertheless, the effect of EO crude fruit extract on iNOS mRNA expression did not reach statistical significance (Figure 4(c)).

### 3.5. Effect of EO Crude Fruit Extract on LPS-Induced Inflammation in BV2 Cells via the ERK and JNK Pathways

**Figure 5:**
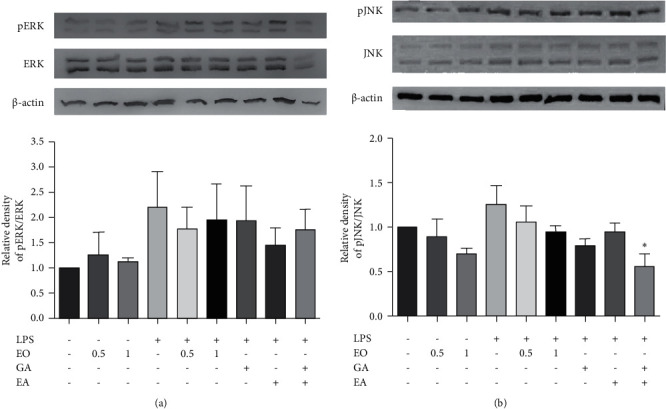
Effect of EO crude fruit extract on the ERK and JNK pathways in BV2 cells. The cells were treated with LPS (1 *μ*g/mL) or EO crude fruit extract (0.5 or 1 *μ*g/mL). After 24 h, cell pellets were collected, and total proteins were extracted. Then, the expression levels of (a) pERK/ERK, (b) pJNK/JNK, and *β*-actin were assessed by Western blot analysis. All data are presented as mean ± SD of three separate experiments. (^*∗*^*p* < 0.05, compared with the LPS-treated group).

Western blot analysis showed the expression levels of both pERK/ERK and pJNK/JNK increased in LPS-inflamed BV2 cells. However, EO crude fruit extract at the concentrations of 0.5 and 1 *µ*g/mL tended to decrease the expression level of pERK/ERK by 19.29% and 11.06% and that of pJNK/JNK by 15.68% and 24.56%, respectively, compared with the LPS-treated group. Nevertheless, EO crude fruit extract effects on either pERK/ERK or pJNK/JNK expression did not reach statistical significance (Figure 5). Interestingly, a combined treatment of EA and GA to the LPS-treated cells significantly decreased the expression level of pJNK/JNK.

### 3.6. Neurite Outgrowth Stimulatory Effects of EO Crude Fruit Extract on LPS-Treated Differentiated Neuro2a Cells

**Figure 6:**
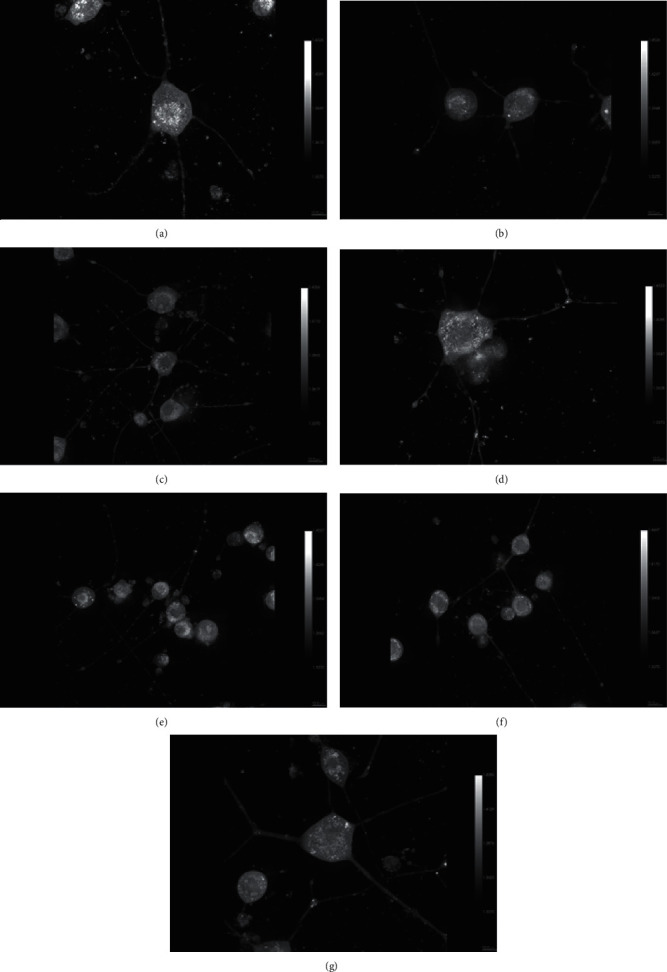
Neurite length of differentiated Neuro2a cells after exposure to EO crude fruit extract, GA, EA, or LPS (×60 magnification). (a) Control; (b) LPS; (c) LPS + EO crude fruit extract 0.5 *μ*g/mL; (d) LPS + EO crude fruit extract 1 *μ*g/mL; (e) LPS + GA; (f) LPS + EA; and (g) LPS + GA + EA.

An earlier study demonstrated that Neuro2a cells expressed TLR4 and LPS administration upregulated inflammatory cytokine expression levels [50]. Therefore, we investigated the direct effect of LPS on neurite length in differentiated Neuro2a cells in this study.  Figure 6 presents the morphology of the differentiated Neuro2a cells. LPS affected neither neurite length nor their morphology.

**Figure 7:**
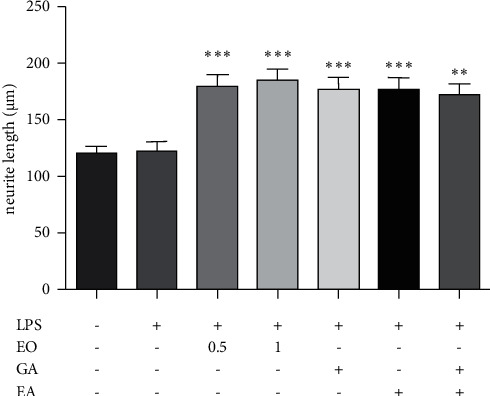
Differentiated Neuro2a cells were treated with LPS (1 *μ*g/mL) or EO crude fruit extract (0.5 or 1 *μ*g/mL) for 24 h. After the treatment, the neurite length was analyzed by MicroCapture v 6.9.10. software (ANOVA; significant at ^*∗∗*^*p* < 0.01 and ^*∗∗∗*^*p* < 0.001, compared with the LPS-treated group).

Figures 6 and 7 show that EO crude fruit extract at the concentrations of 0.5 and 1 *µ*g/mL significantly increased the neurite length of LPS-stimulated differentiated Neuro2a cells by 47.08% and 51.6%, respectively. In addition, GA, EA, and GA + EA treatments significantly induced the neurite length of LPS-treated differentiated Neuro2a cells.

### 3.7. Effect of EO Crude Fruit Extract on the Expression of TuJ1 and MAP2 Neuronal Markers in LPS-Treated Differentiated Neuro2a Cells

**Figure 8:**
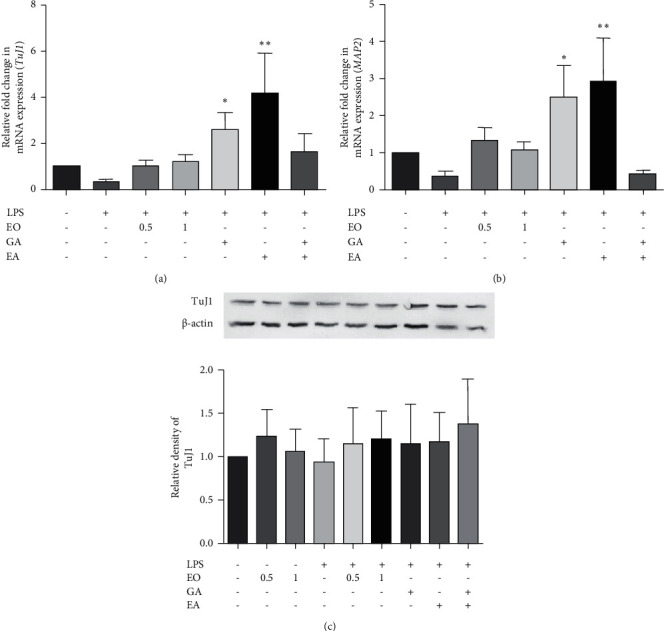
Effect of EO crude extract on the mRNA expression level of two neuronal markers in differentiated Neuro2a cells. Cells were treated with LPS (1 *μ*g/mL) or EO crude fruit extract (0.5 or 1 *μ*g/mL). After 24 h, the cells were collected, and mRNA expression levels of two neuronal markers ((a) TuJ1 gene and (b) MAP2 gene) were tested by RT-qPCR. (c) TuJ1 protein expression was measured by Western blot. The results are presented as mean ± SD. (^*∗*^*p* < 0.05 and ^*∗∗*^*p* < 0.01, compared with the LPS-treated group.)

Figure 8 presents the effects of EO crude fruit extract on the mRNA expression of TuJ1 and MAP2 in Neuro2a cells. In the LPS-treated group, LPS reduced both TuJ1 and MAP2 mRNA expression levels. Interestingly, EO crude extract at 0.5 and 1 *µ*g/mL increased the TuJ1 mRNA expression level by 205.83% and 277.60%, respectively, and the MAP2 mRNA expression level by 290.25% 216.98%, respectively, compared with the LPS-treated group. However, these changes did not reach statistical significance (Figure 8). Single treatments with either GA or EA significantly induced the mRNA expression of both TuJ1 and MAP2.

Interestingly, cell treatment with EO crude fruit extract at the concentrations of 0.5 and 1 *µ*g/mL tended to increase the TuJ1 protein expression level by 27.63% and 18.77%, respectively, compared with the LPS-treated group (Figure 8).

## 4. Discussion

This study evaluated the effect of EO crude fruit extract and its major bioactive compounds against LPS-induced inflammation in both BV2 cells and differentiated Neuro2a cells. The HPLC analysis protocol, modified from Sawant et al. [51], indicated that EO fruit extract contained two major bioactive phenolic compounds, GA and EA. They displayed anti-inflammatory activity by inhibiting LPS-induced NO, PGE-2, and IL-6 production [52]. Thus, they are promising candidates to alleviate various diseases [53, 54]. EA attenuated the levels of both iNOS and MAPK expression levels induced by LPS in microglia [55]. GA can induce neurite outgrowth in neuroblastoma (SH-SY5Y cells) [56]. In addition, both GA and EA were detectable in brain tissues after either oral or intraperitoneal administration, indicating that these substances could pass through the blood-brain barrier [57, 58]. In animal studies, oral administration of 70% ethanol EO crude extract showed analgesic effects on postoperative and neuropathic pain in rat models [59]. Acute and chronic toxicity studies did not show any significant toxic side effects [35, 60, 61].

Interestingly, the combination of EA and GA at the same concentration found in EO crude fruit extract exerted a significantly higher anti‐inflammatory effect than the crude extract *per se*. A variety of phytochemicals present in crude extracts have either antagonistic or synergistic effects. Crude extract has a lower potential than standard treatment, which may be the result of the activity of trace compounds such as alkaloids that are commonly present in the ethanolic extract of EO fruits [62, 63]. Furthermore, alkaloids have display antagonistic effects to phenolic compounds during antioxidant activity [64]. Further studies are needed to explore the bioactivity-guided fractionation of EO extract that possesses anti-inflammatory and neuroprotective activities.

Recently, LPS detection in the human body was associated with several inflammatory-related diseases. An earlier report showed that LPS could be detected in the cerebrospinal fluid (2.5 *μ*g/L) of patients with a meningococcal disease [65]. It induces neuroinflammation as a bridge in neurodegenerative disease models [66]. LPS was also reported to induce microglia activated through the MAPK pathway [55, 67] that often precedes neuronal death [14]. In addition, a study dealing with LPS-induced cognitive impairment and neuroinflammation showed the interconnection of microglia activation and neuronal cell loss with increased NO and TNF-*α* production [15]. Inflammatory neurodegeneration involved activated microglia and overexpression of both iNOS and NO, causing toxicity to neurons [17, 52]. Our study demonstrates the anti-inflammatory effect of EO crude fruit extract reflected in decreased levels of released NO, IL-6, and TNF-*α* into the culture media and in downregulation of the expression levels of iNOS mRNA. A recent study also showed anti-inflammatory effects of EO extract in reducing NO, IL-6, and TNF-*α* levels in macrophages [68]. Moreover, other investigations that involved neuroinflammation demonstrated the effectiveness of both GA and EA to inhibit iNOS protein expression in either glia or neuronal cells [41, 46, 69].

In the AD brain, amyloid beta can activate microglia through TLR, resulting in increased IL-6 and NO production [70]. MAPK signaling pathways are implicated in AD development, and persistent activation of JNK signaling pathways might mediate neuronal apoptosis in AD [71]. The study on phenolic compounds demonstrated that vanillic acid improved memory function by inhibiting the JNK signaling pathway during LPS-induced neurotoxicity in the mouse model [72]. Moreover, a morphology study on human-induced pluripotent stem cells of AD neurons indicated shorter neurites and decreased branching with altered synaptic function [73]. In the present study, EO crude fruit extract significantly decreased IL-6, NO, and TNF-*α* production, partly due to the suppression of both ERK and JNK signaling pathways in LPS-treated BV2 cells. The MAPK pathway regulates many aspects of the proinflammatory cytokine synthesis, including NO, IL-6, and TNF-*α* [18, 19]. Suppression of the MAPK pathway reportedly reduced neuroinflammation and neurodegenerative disorders [20, 21]. Moreover, we found that LPS did not significantly affect the activation of ERK and JNK in Neuro2a cells (data not shown).

In the present study, LPS-induced NO, IL-6, and TNF-*α* production in BV2 cells but did not activate NO and IL-6 in differentiation Neuro2a cells. These different response patterns to LPS-induced inflammation may result from different specific receptors present on the cell surface. BV2 cells have higher expression levels of IL-6 and TLR4 than Neuro2a cells [74]. Neuroblastoma cells can differently express those receptors. For instance, the SHSY-5Y cell line did not show any TLR4 expression [75]. The NB-1 cell line has been proved to express TLR4 intracellularly but has not responded to LPS due to the lack of interferon regulatory factors [76]. However, the capability of LPS to induce NO production was observed in differentiated Neuro2a cells treated with a high concentration (12.5 *μ*g/mL) of LPS [77]. Our study showed that basal IL-6 levels in differentiated Neuro2a cells were higher than those of BV2 cells, while NO levels were lower. Several neuroblastoma cells are capable of producing IL-6 [78, 79] spontaneously. IL-6 has a notorious role in adult neuronal differentiation during the development of new neuronal and glial cells from neural stem cells (NSCs) [16]. Our study showed that EO fruit extract significantly decreased the IL-6 production in differentiated Neuro2a cells. Consistently, the extract significantly stimulated neurite outgrowth in differentiated Neuro2a cells and potentiated the TuJ1 and MAP2 mRNA expressions and the TuJ1 protein expression. Both GA and vanillic acid have been shown to possess the ability to promote neurite outgrowth from hippocampal neurons [22]. Neurite outgrowth is crucial for neuronal plasticity, regeneration, and synapticity [80]. The length of neurite outgrowth, which depends on microtubule formation, is the most commonly used parameter in assessing whether studied compounds can affect neurite growth [81]. Histopathological examination of the brain tissue in AD showed significantly lower neurite outgrowth [82] and dramatically decreased the mature neuronal marker MAP2 expression [83]. Specific drugs used in AD, such as memantine and donepezil, reportedly enhance neurite outgrowth in primary cortical neurons to improve cognitive function [84]. However, the molecular mechanism of EO's stimulatory effect on neurites needs to be elucidated.

We observed two main limitations of this study. First, the experiments were independently performed using only BV2 cells and Neuro2a cells, representing microglia and neuroblastoma cells. However, the effect of EO fruit extract was not investigated in other types of neuronal cells of the CNS, such as astrocytes and oligodendrocytes. Second, the Neuro2a cell line, being of the neuroblastoma origin, is useful for evaluating only some neuronal properties that may differ in their pharmacological and functional characteristics from neuronal cells [85].

**Figure 9:**
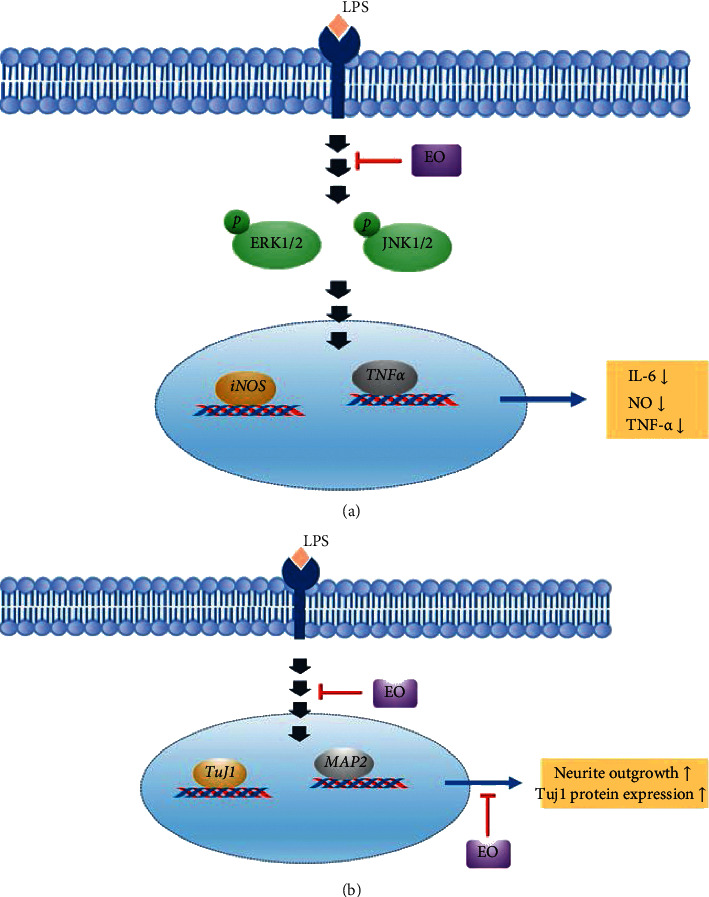
Proposed pathways involved in neuroprotective and anti-inflammatory activities of *Emblica officinalis*. (a) EO crude fruit extract significantly attenuated proinflammatory cytokine IL-6, NO, and TNF-*α* in LPS-induced BV2 cells via the ERK1/2 and JNK signaling pathways. (b) Neuroprotective effects are associated with enhanced neurite outgrowth that the TuJ1 and MAP2 mRNA expressions may influence.

Neuroinflammation stimulated by LPS in BV2 cells caused an increase in IL-6, NO, and TNF-*α* production. These findings imply that EO crude fruit extract can ameliorate neuroinflammation in BV2 cells by reducing IL-6, NO, and TNF-*α* production, possibly via the suppression of ERK and JNK signaling pathways. In addition, EO crude fruit extract was showed to have neuroprotective effects by enhancing neurite outgrowth in Neuro2a cells and may also take part in the upregulation of TuJ1 and MAP2 mRNA expressions as well as TuJ1 protein expression (Figure 9). GA and EA, found in the extract, can serve as anti‐inflammatory compounds that exhibit inhibitory effects through the MAPK pathway [55, 86]. Complementary alternative medicines can improve neuronal function by enhancing neurite outgrowth and reducing microglia activation [87, 88]. To the best of our knowledge, this is the first report on antineuroinflammation in microglia and neurite outgrowth that are promoted by the activity of ethanolic EO crude fruit extract. It is essential for EO crude fruit extract and its bioactive compounds to be further investigated toward clarifying their potential role in decreasing inflammatory processes and enhancing neurite outgrowth in neuroinflammatory diseases.

## 5. Conclusions

EO crude fruit extract could decrease neuronal inflammation in an LPS-activated model by reducing proinflammatory cytokines released from microglia activation. Our study provides the first evidence that EO fruit extract effectively enhanced neurite length growth by increasing neuronal marker expression and decreasing neuroinflammation *in vitro*. However, possible underlying mechanisms and molecular targets should be further investigated, contributing to the clinical application of EO crude fruit extract or its derivatives in the therapy of neuroinflammatory diseases. In addition, future studies should fully understand the cellular mechanisms involved in neuroprotection in the coculture model.

## Figures and Tables

**Table 1:** Primer sequences of *β*-actin, TuJ1, MAP2, GAPDH, iNOS, and TNF-*α*.

Gene name	Forward/reverse primer sequence
TuJ1	(F) 5′-ACCCCGTGGGCTCAAAAT-3′
	(R) 5′-CCGGAACATGGCTGTGAACT-3′

MAP2	(F) 5′′-CCTGGTGCCCAGTGAGAAGA-3′
	(R) 5′′-GTCCGGCAGTGGTTGGTTAA-3′

GAPDH	(F) 5′-CTCGTGGAGTCTACTACTGGTGT-3′
	(R) 5′-GTCATCATACTTGGCAGGTT-3′

iNOS	(F) 5′-ATGAGGTACTCAGCGTCGTCCAC-3′
	(R) 5′-CCACAATAGTACAATACTACTTGG-3′

## Data Availability

All the data used to support the findings of the study are available from the corresponding author upon request.

## Abbreviations

AD: Alzheimer's disease
ANOVA: Analysis of variance
DMEM: Dulbecco's modified Eagle's medium
EA: Ellagic acid
ELISA: Enzyme-linked immunosorbent assay
ERK: Extracellular signal-regulated kinases
EO: *Emblica officinalis*
FBS: Fetal bovine serum
GA: Gallic acid
HPLC: High-performance liquid chromatography
JNK: c-Jun N-terminal kinases
IL-6: Interleukin-6
iNOS: Inducible nitric oxide synthase
LPS: Lipopolysaccharide
MAP2: Microtubule-associated protein 2
MAPK: Mitogen-activated protein kinase
MEM: Minimal essential medium
NED: *N*-(1-naphthyl) ethylenediamine
NO: Nitric oxide
PD: Parkinson's disease
PVDF: Polyvinylidene fluoride
RIPA: Radioimmunoprecipitation assay
ROS: Reactive oxygen species
RPMI: Roswell Park Memorial Institute
RT-qPCR: Reverse transcription-quantitative polymerase chain reaction
SDS: Sodium dodecyl sulfate
TMB: Tetramethylbenzidine
TNF-*α*: Tumor necrotic growth factor-*α*
TLR: Toll-like receptor
XTT: 2,3-Bis-(2-methoxy-4-nitro-5-sulfophenyl)-2H-tetrazolium-5-carboxanilide.
